# Characterizing the experience of agitation in patients with bipolar disorder and schizophrenia

**DOI:** 10.1186/s12888-018-1673-3

**Published:** 2018-04-16

**Authors:** Jenna Roberts, Alfredo Gracia Canales, Sophee Blanthorn-Hazell, Anca Craciun Boldeanu, Davneet Judge

**Affiliations:** 1 Adelphi Real World, Macclesfield, UK; 2 Patient Advocacy Ferrer, Barcelona, Spain; 3 Scientific Department, Ferrer, Barcelona, Spain

**Keywords:** Schizophrenia, Bipolar disorder, Agitation, Patient survey

## Abstract

**Background:**

Agitation is a common manifestation of bipolar disorder and schizophrenia which includes symptoms ranging from inner tension and unease to violence and aggression. Much of the existing literature has focused on agitation in the acute setting, with the patient experience poorly defined. Thus, the aim of this study was to characterize agitation and its management from a patient perspective, with the focus on those who reside in the community.

**Methods:**

Surveys were completed across Germany, Spain and the UK by 583 community dwelling patients with schizophrenia or bipolar disorder who experienced episodes of agitation. Patients were recruited via either their physician or through patient support groups. The survey captured information on demographics, disease characteristics, frequency of agitation episodes and different pre-defined severity levels ranging from mild to severe, symptoms experienced during an episode, awareness of agitation and coping strategies employed by the patient. Statistics were descriptive in nature.

**Results:**

The most commonly reported symptoms during an episode of agitation were feeling uneasy (*n* = 373, 64%), restless (*n* = 368, 63%) or nervous (n = 368, 63%). Patients experienced an average of 22.4 (SD 57.2) mild, 15.4 (SD 61.2) moderate, 6.8 (SD 63.3) moderate-intense and 2.9 (SD 24.4) severe episodes within the last 12 months; on average 2.7 (SD 6.8) required hospital attendance. Half of patients (*n* = 313) had attended hospital due to agitation. In total, 71% of patients (*n* = 412) were aware they were becoming agitated either always or sometimes and 61% of patients (*n* = 347) were aware of agitation triggers either always or sometimes. The majority of patients reported being able to sometimes control their agitation (56%, *n* = 329) but 16% (*n* = 94) stated that there is typically nothing they can do. To cope with episodes 55% (*n* = 125) of schizophrenia patients and 66% (*n* = 234) of bipolar disorder patients reported taking prescribed medication.

**Conclusion:**

Community based patients with schizophrenia and bipolar disorder reported frequently experiencing agitation episodes which they defined most commonly as feeling uneasy, restless or nervous. A range of coping strategies were reported but they were not always successful, highlighting an area of unmet need in this population.

**Electronic supplementary material:**

The online version of this article (10.1186/s12888-018-1673-3) contains supplementary material, which is available to authorized users.

## Background

Agitation is a common manifestation in a number of psychiatric conditions and in particular, dementia, bipolar disorder and schizophrenia [1, 2]. It has been described as a common, but often unaddressed problem in psychiatry [3]. Currently, there is no universally accepted definition as to what constitutes as agitation, [4] although recent attempts have been made to reach a consensus for agitation in dementia and cognitively impaired patients [1]. In schizophrenia and bipolar, a broad range of features have been described including physical or mental unease, inner tension, restlessness, irritability, excitement, uncooperativeness, anxiety, motor activity that is excessive, inappropriate or purposeless, and motor tension [3–6]. Importantly, although aggressive and violent behavior sometimes occur, expert opinion asserts that agitation is distinct from aggression [1]. Further complicating the detection of agitation is the occurrence of akathesia, i.e. drug-induced restlessness which can arise from the anti-psychotic medication that is often used to treat schizophrenia and bipolar disorder [7].

Recommendations have been made for how agitation should be assessed and managed in psychiatric patients [8]. Current treatment includes pharmacological approaches, typically with antipsychotics or benzodiazepines and parenteral formulations are often used during acute episodes to facilitate rapid drug action [9]. Non pharmacological de-escalation strategies are also advocated as a first line approach including talking therapies and provision of a safe, low stimulus environment [4]. Physical restraint and isolation are sometimes required but are expected to be a last resort [8, 10].

The extent to which long term control over agitation is achieved for patients with schizophrenia and bipolar disorder is largely unknown. Much of the existing literature has focused on acute episodes in clinical settings and particularly emergency departments [11] and economic analysis has shown that agitation contributes significantly to direct medical costs in these settings [12]. However, few studies have considered the day to day experience of agitation for patients who are community dwelling [3, 4]. In general, the patient’s perspective has been largely overlooked. For example, although a range of tools for measuring agitation have been developed and validated, (e.g. the Positive and Negative Syndrome Scale Excited Component subscale [PANSS-EC] the Modified Overt Aggression Severity Scale, [MOASS], the Overt Agitation Severity Scale [OASS] and the Behavioral Activity Rating Scale[BARS]) [13–16] they are clinician-reported rather than patient-reported outcome measures. As a consequence of this, these measures focus more on observable behaviors such as uncooperativeness or violence. However, the more subtle internal processes associated with agitation, particularly at the milder stages, may be equally important and may have a substantial impact on the patient’s overall ability to manage their condition.

The present work sought to better understand the experience of agitation for patients with schizophrenia and bipolar disorder. Specifically, the focus of this work is on the patient’s perspective and the way agitation manifests for those who are community dwelling and therefore at a relatively more quiescent disease stage. We explored the way in which patients describe agitation, the frequency with which this symptom is experienced and the steps patients take to try and manage this symptom using a multi-country survey based approach.

## Methods

This was a cross-sectional survey conducted in Germany, Spain and UK of patients diagnosed with bipolar disorder or schizophrenia. Where possible and if applicable, additional data were also collected from the patient’s caregiver via a caregiver questionnaire. These data are reported elsewhere. The survey was conducted between October 2016 – January 2017 and all data collected were fully anonymized. Patient support groups in each country reviewed and provided input into the content of the survey. The study was also reviewed and approved by an international ethics committee for centralised methodological ethics approval.

### Sample

Patients were identified either by their physician or through a local patient support group and could either complete the survey online or in pen/paper format.

### Inclusion criteria

To be eligible to participate, patients were required to be ≥18 years old, be community dwelling, have a diagnosis of bipolar or schizophrenia and state that they had experienced at least one episode of agitation in the last 12 months for which they had sought professional help. Patients who were currently involved in a clinical trial were ineligible. Participation was entirely voluntary and participants were free to withdraw at any time without providing a reason.

### Survey instrument

The survey used in this research is included in the Additional file 1. A broad definition of agitation was provided at relevant points throughout the survey. The definition was designed to ensure some consistency but without being overly restrictive or biasing responses. The definition stated that: *“When people experience an episode of agitation they may feel noticeably more tense, restless, wound up, uneasy or short-tempered than usual. Some people talk a lot more than they would usually or find it difficult to keep still. Sometimes agitation leads to violent or aggressive behavior but this isn’t always the case. An episode of agitation will subside after a while.”*

### Demographics

The initial section of the survey collected demographic information and basic information about the patients’ condition including age, gender, employment status, living circumstances, whether the primary diagnosis was bipolar disorder or schizophrenia and current disease severity ranging from mild to severe. The following definitions were used for severity: very mild - overall, my mental health disorder is manageable and does not have a major impact on my quality of life, mild - my mental health disorder is manageable most of the time but has had an impact on my quality of life, moderate - my mental health disorder is sometimes manageable but I also have periods where things are very difficult and severe - my mental health disorder is difficult to manage and has had a significant impact on my quality of life.

### Characterizing agitation

A predefined list of symptoms that might be associated with agitation was included in the survey based on a review of the published literature, including descriptors that feature in the available clinician-reported outcome measures (e.g. PANSS-EC). Patients were asked to state how often they had felt each of these symptoms in association with agitation over the past year. Five response options were available including never, sometimes, occasionally, often and always.

In order to estimate the frequency of agitation episodes, patients were asked to report, from the previous year, how many episodes of mild-moderate agitation episode, moderate-intense episodes and severe episodes they had experienced. The following definitions were provided: *Mild feelings are easier to control or ignore. Moderate feelings are often harder to control and may get in the way of day-to-day life. Moderate-intense feelings seriously disrupt and interfere with your day-to-day-life. Severe feelings are almost impossible to control and may lead to more serious outcomes like violent behaviour or having to go to hospital.* If patients had experienced at least one agitation episode at a given severity level, they were asked to indicate how many of these episodes had required help from a doctor or nurse and how many had required hospital attendance. If patients had been to hospital, they were asked how many days they experienced feeling agitated before going.

### Awareness of agitation

Two questions explored patient awareness of agitation. Patients were asked if they were aware when they were becoming agitated and also if they were aware of specific things that triggered agitation in themselves. For both questions, there were three response options: always/most of the time, sometimes and rarely/ never.

### Coping with agitation

Attempts to cope with agitation were assessed in a number of ways. First, patients were asked which of the following statements they agreed with to determine coping abilities: *1. When I feel agitated, there is normally nothing I can do, I just have to wait for it to pass, 2. When I feel agitated, there are some techniques I can try but they are not very effective, 3. When I feel agitated, I can sometimes control it but other times I cannot, 4. When I feel agitated, I can usually control it quite well.*

Following this, from a predefined list, patients were asked which specific coping strategies they had tried over the previous year in response to an agitation episode (e.g. “do something I find relaxing…”). The list also included the option to select “nothing/ wait for it to pass.” For each item selected, patients were asked whether or not the technique had helped.

Further questions asked patients if they had been prescribed medication that they used “as and when needed” at home to help cope with episodes of agitation (yes/no) and if yes, they were asked to specify the drug (if known) and the level of satisfaction with a.) how effective the medication was in reducing agitation and b.) how satisfied they were with how long the medication typically takes to work. Both satisfaction questions were addressed with a five point scale ranging from very satisfied to very dissatisfied.

### Statistical analyses

The study was designed to be descriptive only with no formal hypothesis testing. The reported statistics depended on the type of variable described: for numeric variables, the mean and standard deviation are reported, for categorical variables the number and percentage are shown. Missing data were removed from the specific analysis. However, participants removed from one piece of analysis were still eligible for inclusion in other analyses. All analysis was performed with IBM® SPSS® Data Collection Survey Reporter Version 7.

## Results

A total of 583 patients were surveyed across 3 countries: Germany (*n* = 202), Spain (*n* = 200) and the UK (*n* = 181), respectively.

### Patient demographics

Patient demographics are described in Table 1. Overall there was an equal split between male and females (males 48%) and patients had a mean age of 42 years. A slightly higher proportion of the sample had a primary diagnosis of bipolar disorder (61%) relative to 39% with schizophrenia. The majority of patients were unemployed or retired (*n* = 322, 55%) and of these patients, 71% stated that they were out of work as a result of their condition. Most patients were currently living with someone, typically their spouse or another family member and only 92 patients sampled (16%) lived alone. Disease severity as reported by the patient was most commonly rated as moderate (*n* = 250, 43%).

**Table 1:** Patient Demographics

Patient demographics	Total Sample (*n* = 583)	Schizophrenia	Bipolar
Age: mean (SD)	42.0 (12.6)	40.3 (13.4)	43.1 (12.0)
Gender: % male	281 (48%)	142 (62%)	139 (39%)
Condition			
Bipolar disorder	354 (61%)	–	354 (100%)
Schizophrenia	229 (39%)	229 (100%)	–
Employment status			
Fulltime	97 (17%)	18 (8%)	79 (22%)
Part time	81 (14%)	32 (14%)	49 (14%)
Out of work	406 (70%)	179 (78%)	227 (64%)
Living circumstances			
Partner/ spouse	229 (39%)	46 (20%)	183 (52%)
Other family member	144 (25%)	74 (32%)	70 (20%)
Mean time since diagnosis, years (SD)	13.4 (9.54)	14.4 (9.30)	12.8 (9.65)
Current severity of condition			
Very mild	38 (7%)	13 (6%)	25 (7%)
Mild	168 (29%)	69 (30%)	99 (28%)
Moderate	250 (43%)	100 (44%)	150 (42%)
Severe	127 (22%)	47 (21%)	80 (23%)

### Characterizing agitation

**Fig. 1:**
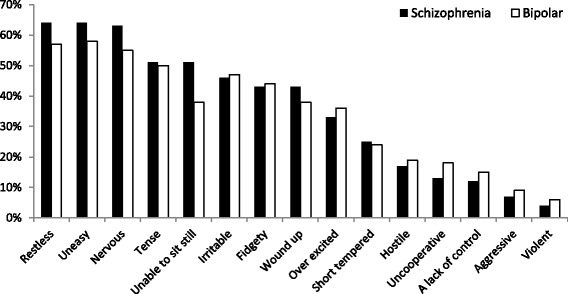
Symptoms experienced during an episode of agitation in the last 12 months. Self-reported symptoms experienced in the last 12 months split by primary diagnosis of schizophrenia or bipolar disorder

The feelings and symptoms that patients reported experiencing during an episode of agitation are depicted in Fig. 1. The percentages displayed are the proportion of patients who stated that they ‘always’ or ‘often’ experience this symptom as part of an episode of agitation. The most common symptoms reported were feeling uneasy (*n* = 373, 64%), restless (*n* = 368, 63%) or nervous (*n* = 368, 63%). Violence and aggression were the least common symptoms reported. Minimal differences were observed depending on whether the patient had a primary diagnosis of bipolar disorder.

Patients experienced an average of 22.4 (SD 57.2) mild, 15.4 (SD 61.2) moderate, 6.8 (SD 63.3) moderate-intense and 2.9 (SD 24.4) severe episodes in the last 12 months, of which 13%, 29%, 40% and 55% of these episodes required assistance from a healthcare professional respectively. Overall, a mean number of 2.7 (SD 6.8) episodes required hospital attendance. In total, 313 patients had been to their hospital or clinic due to agitation and reported experiencing agitation symptoms for a mean of 12 days (SD 25.08) prior to going to hospital based on the last time this happened.

### Awareness and recognition of agitation

**Fig. 2:**
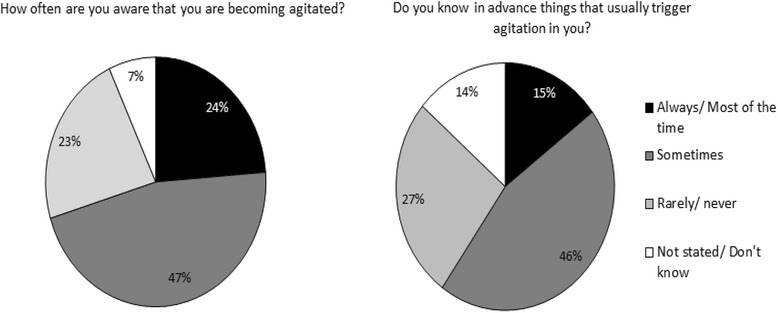
**a** and **b**: Patient awareness of when they are becoming agitated and triggers of their agitation. Self-reported patient awareness of when they are becoming agitated (**a**) and awareness around triggers of agitation episodes (**b**). The question asked to patients who completed the survey is shown above each pie chart

Figure 2a and b show how often patients are aware when they are becoming agitated and how often they know in advance things that trigger an episode of agitation. In total, 71% of patients (*n* = 412) were aware they were becoming agitated either always or sometimes and 61% of patients (*n* = 347) were aware of agitation triggers either always or sometimes.

### Coping with agitation

When patients were asked whether or not they can usually control their agitation, 12% of patients (*n* = 71) reported that they can usually control their agitation quite well, 15% (*n* = 89) said there were techniques they try but they were not very effective, the majority (56%) (*n* = 329) stated they can sometimes control agitation and 16% (*n* = 94) stated that there is normally nothing they can do.

**Fig. 3:**
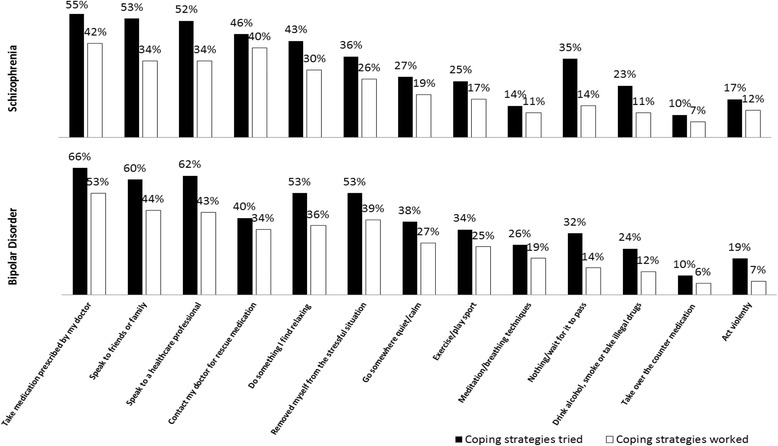
Coping strategies tried by patients and their helpfulness. Self-reported coping strategies used by patients during an episode of agitation, and those reported to have worked to some extent by the patients that tried them. Split by primary diagnosis of schizophrenia or bipolar disorder

The coping strategies that patients had employed, and whether these had been effective are shown in Fig. 3. Most commonly, patients took medication that had been prescribed by their physician. Talking to family and friends and talking to a healthcare professional was also a common strategy. Patients did also report negative coping behaviours such as acting violently or drinking alcohol/ smoking/ taking illegal drugs. In total, 53% (*n* = 189) of bipolar patients reported that taking medication as a coping technique had helped. Aside from this, all attempted strategies were reported as helping by fewer than 50% of patients. In addition, 35% of schizophrenia patients (*n* = 113) and 32% of bipolar patients (*n* = 80) said that they often just did nothing. Specifically around medication as an approach for coping with agitation, patients most commonly reported taking diazepam for this purpose (*n* = 150, 48%) (however, results should be interpreted with caution due to lack of confirmation by a physician). Of the patients who had been prescribed short term medication to help with agitation, 74% (*n* = 228) and 61% (*n* = 191) were satisfied with the impact of the medication on agitation and with the onset time for symptoms management.

## Discussion

The results from this European survey demonstrate that for many bipolar and schizophrenia patients, feeling agitated is a common occurrence, particularly at mild to moderate severity levels, with patients reporting a mean of 22.4 episodes of mild agitation per year and 15.4 moderate episodes. The experience of agitation was associated with internal feelings (e.g. feeling tense, restless and uneasy) more commonly than more overt behaviors such as aggression. Indeed recent qualitative research with healthcare professionals described a range of three agitation states and only the most severe level included aggressive behavior [6]. However, it is possible that the patients who are more likely to experience violent and aggressive forms of agitation were underrepresented in the survey, due to a lack of willingness to participate. Nonetheless, the data highlight the importance of more subtle symptoms for many patients which represent a potentially unseen psychological burden. Healthcare professionals will need to be aware of these features in order to identify patients suffering from agitation and act accordingly. This may require a shift in thinking given that physicians have typically focused more on more severe episodes and observable features such as excess motor activity [1]. However, this view may have led to an overly restrictive understanding of agitation and an underestimation of the extent of the problem at more mild to moderate levels as experienced by the community dwelling patients sampled here. In line with this, to date, much previous research and published treatment guidelines have focused on acute episodes of severe agitation, particularly in emergency room settings [10, 17, 18]. For example, San et al. reported that 4.6% of psychiatric emergencies in the emergency room or inpatient settings featured agitation [17]. Better understanding and management of mild to moderate agitation could potentially reduce the frequency of these more serve episodes.

Approximately two thirds of patients showed good awareness of agitation, both in terms of knowing when they were experiencing symptoms and knowing specific triggers (situations, events, people) that lead them to experience an agitation episode. Although this awareness did not apply to all patients or all agitation episodes, the data suggests that at least some of the time, patients may be able to self-identify when they start to experience problems. It would be at this point when early detection and intervention might be possible. With the right tools, this could prevent further escalation of the agitation episode. Similarly, in the current study, we found that when patients attended their hospital or clinical because of agitation, on average, they had waiting 12 days. This demonstrates a clear unmet need and window of opportunity where de-escalation techniques that might be used to avoid the need for the patient to present at hospital. This in turn may support avoidance of some of the high inpatient costs associated with agitation that have previously been described [12].

Indeed, the data revealed that patients are already attempting a range of techniques to try to cope with agitation, including taking their medication or speaking to family/ friends or their physician. However, the techniques are not always effective and around a third of patients reported that they often did nothing and just waited for the episode to pass. Use of “as needed” medication was reported by approximately half the sample but for these patients, overall satisfaction with the reduction of agitation and the onset time was fairly high, supporting the value of a pharmacological intervention for some patients. It is unlikely that any single approach will be successful all of the time but there is a clear need for more effective management strategies targeted towards reducing the frequency and severity of agitation episodes.

Taken together, the data indicate that patients are aware of their agitation and the majority take steps to try and self-manage. This should be encouraged, with the World Health organization listing involvement in personal care plans and treatment decisions as key aspects of patient empowerment [19]. The present study has described the experience of agitation specifically from the patient’s perspective whereas previous research has focused more on observable manifestations of agitation and the perspective of the clinician. Further research is needed to understand the epidemiology of agitation in the community setting, as well as the burden to the patient and the impact on the healthcare system. Development of validated Patient Reported Outcome (PRO) tools would facilitate observational research and would contribute further to understanding agitation from the patient’s view.

### Limitations

The patients who were willing to take part in the survey may not be representative of the broader bipolar and schizophrenia population. In particular, severe patients who may be too unwell to complete the survey are unlikely to be captured in the present study and we also excluded anyone currently being treated in an inpatient setting. Inclusion criteria also necessitated that patients had experienced agitation and sought professional help for this at some point within the past 12 months. Consequently, the study has focused on a subpopulation of patients known to experience problems with this specific symptom. Data were not collected from the treating physician or any other healthcare professional, meaning that objective clinical information from the patient’s medical records (e.g. medication status) were not captured and, therefore, limited data are available to provide insight into the relationship between agitation and clinical characteristic. All data were obtained from the patients themselves and recall accuracy and bias are therefore a limitation. In particular, a number of questions in the survey asked patients to think about their experience of agitation over the past year. While some patients may have had recent episodes to draw on when answering these questions, others may have to have relied on their memory of agitation that was further in the past. In addition, the present study was unable to use validated patient-reported outcome measures due to lack of availability. The development of such instruments would be of significant value for facilitating further research in this area.

## Conclusions

Amongst the patient’s sampled, mild and moderate agitation were a common experience which patients described as involving feelings of unease, restlessness and nervousness. These feelings may not always be obvious to external observers so management is reliant on good communication between patients and the healthcare teams involved in their care. Patients were attempting a range of coping techniques but these were not fully effective, highlighting an area of unmet need. Nevertheless, the data supports the fact that patients do talk to their doctor about this symptom. Patients in this study are assumed to be relatively well; many had social support and were currently living with family, 30% were in employment, all were outpatients and all were willing and able to complete the questionnaire measures. In light of this, it is significant that agitation was commonly experienced and the data here may underestimate the true extent of the problem.

## Additional file

Additional file 1: “Patient questionnaire v10.1 05.09.16”. Questionnaire administered to patients to characterize their experience of agitation, entitled “Self-completion Form for Patients”. (PPTX 147 kb)

## Abbreviations

BARS: Behavioral Activity Rating Scale
MOASS: Modified Overt Aggression Severity Scale,
OASS: Overt Agitation Severity Scale
PANSS-EC: Positive and Negative Syndrome Scale Excited Component
SD: Standard Deviation
UK: United Kingdom

## References

[1] Cummings J, Mintzer J, Brodaty H, Sano M, Banerjee S, Devanand DP (2015). Agitation in cognitive disorders: international psychogeriatric association provisional consensus clinical and research definition. Int Psychogeriatr.

[2] Mintzer JE (2006). Introduction: the clinical impact of agitation in various psychiatric disorders: management consensus and controversies. J Clin Psychiatry..

[3] Sachs G (2006). A review of agitation in mental illness: burden of illness and underlying pathology. J Clin Psychiatry.

[4] Marder SR (2006). A review of agitation in mental illness: treatment guidelines and current therapies. J Clin Psychiatry..

[5] Yu J, Cheung J. A new health-related quality of life instrument for leukemia: will it be widely adopted soon? Drugs Context. 2013;2013 10.7573/dic.212253. 10.7573/dic.212253 PMC3884751 24432041

[6] Rubio-Valera M, Huerta-Ramos E, Baladón L, Aznar-Lou I, Ortiz-Moreno JM, Luciano JV (2016). Qualitative study of the agitation states and their characterization, and the interventions used to attend them. Actas Esp Psiquiatr.

[7] Poyurovsky M (2010). Acute antipsychotic-induced akathisia revisited. Br J Psychiatry.

[8] Garriga M, Pacchiarotti I, Kasper S, Zeller SL, Allen MH, Vázquez G (2016). Assessment and management of agitation in psychiatry: expert consensus. World J Biol Psychiatry.

[9] Mohr P, Pecenák J, Svestka J, Swingler D, Treuer T (2005). Treatment of acute agitation in psychotic disorders. Neuro Endocrinol Lett.

[10] Zeller SL, Citrome L (2016). Managing agitation associated with schizophrenia and bipolar disorder in the emergency setting. West J Emerg Med.

[11] Marco CA, Vaughan J (2005). Emergency management of agitation in schizophrenia. Am J Emerg Med.

[12] Serrano-Blanco A, Rubio-Valera M, Aznar-Lou I, Baladón Higuera L, Gibert K, Gracia Canales A (2017). In-patient costs of agitation and containment in a mental health catchment area. BMC Psychiatry.

[13] PANSS-EC in Acute Psychosis and Agitation in a Psychiatric ER. Medscape. https://www.medscape.com/viewarticle/744430_1. Accessed 9 Sep 2017.

[14] Alderman N, Knight C, Morgan C (1997). Use of a modified version of the overt aggression scale in the measurement and assessment of aggressive behaviours following brain injury. Brain Inj.

[15] Yudofsky SC, Kopecky HJ, Kunik M, Silver JM, Endicott J (1997). The overt agitation severity scale for the objective rating of agitation. J Neuropsychiatry Clin Neurosci.

[16] Swift RH, Harrigan EP, Cappelleri JC, Kramer D, Chandler LP (2002). Validation of the behavioural activity rating scale (BARS): a novel measure of activity in agitated patients. J Psychiatr Res.

[17] San L, Marksteiner J, Zwanzger P, Figuero MA, Romero FT, Kyropoulos G (2016). State of acute agitation at psychiatric emergencies in Europe: the STAGE study. Clin Pract Epidemiol Ment Health CP EMH.

[18] Allen MH, Currier GW, Carpenter D, Ross RW, Docherty JP. The expert consensus guideline series. Treatment of behavioral emergencies 2005. J Psychiatr Pract. 11 Suppl 1. https://www.ncbi.nlm.nih.gov/pubmed/16319571. Accessed 9 Sep 2017. 10.1097/00131746-200511001-00002 16319571

[19] Organization WH, others. User empowerment in mental health: a statement by the WHO Regional Office for Europe-empowerment is not a destination, but a journey. 2010. http://apps.who.int/iris/bitstream/handle/10665/107275/E93430.pdf;jsessionid=6BB383B33AB6838E511F2AAB34329C08?sequence=1. Accessed 22 Sep 2017.

