# Supplementary material for: Characterizing the experience of agitation in patients with bipolar disorder and schizophrenia
Source: BMC Psychiatry. 2018 Apr 16;18:104. doi: 10.1186/s12888-018-1673-3 (PMC5902921; doi:10.1186/s12888-018-1673-3)
Supplement: Supplementary file 1 — “Patient questionnaire v10.1 05.09.16”. Questionnaire administered to patients to characterize their experience of agitation, entitled “Self-completion Form for Patients”. (PPTX 147 kb) [file 12888_2018_1673_MOESM1_ESM.pptx]

## Slide 1
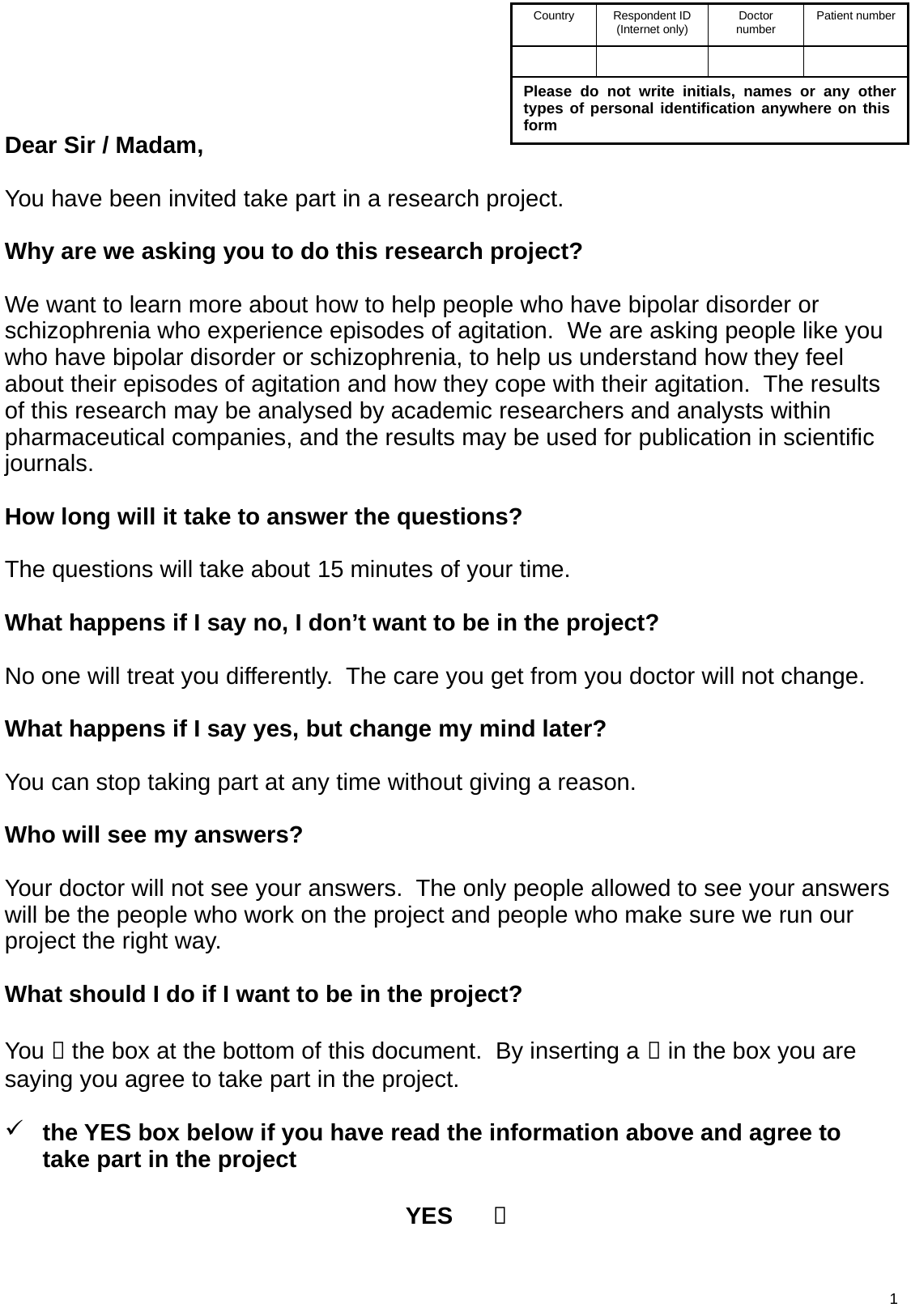

| Country | Respondent ID (Internet only) | Doctor number | Patient number |
| --- | --- | --- | --- |
| | | | |
| Please do not write initials, names or any other types of personal identification anywhere on this form | | | |
| Dear Sir / Madam, You have been invited take part in a research project. Why are we asking you to do this research project? We want to learn more about how to help people who have bipolar disorder or schizophrenia who experience episodes of agitation. We are asking people like you who have bipolar disorder or schizophrenia, to help us understand how they feel about their episodes of agitation and how they cope with their agitation. The results of this research may be analysed by academic researchers and analysts within pharmaceutical companies, and the results may be used for publication in scientific journals. How long will it take to answer the questions? The questions will take about 15 minutes of your time. What happens if I say no, I don’t want to be in the project? No one will treat you differently. The care you get from you doctor will not change. What happens if I say yes, but change my mind later? You can stop taking part at any time without giving a reason. Who will see my answers? Your doctor will not see your answers. The only people allowed to see your answers will be the people who work on the project and people who make sure we run our project the right way. What should I do if I want to be in the project? You  the box at the bottom of this document. By inserting a  in the box you are saying you agree to take part in the project. the YES box below if you have read the information above and agree to take part in the project YES  | | |
| --- | --- | --- |
| | | |
1

## Slide 2
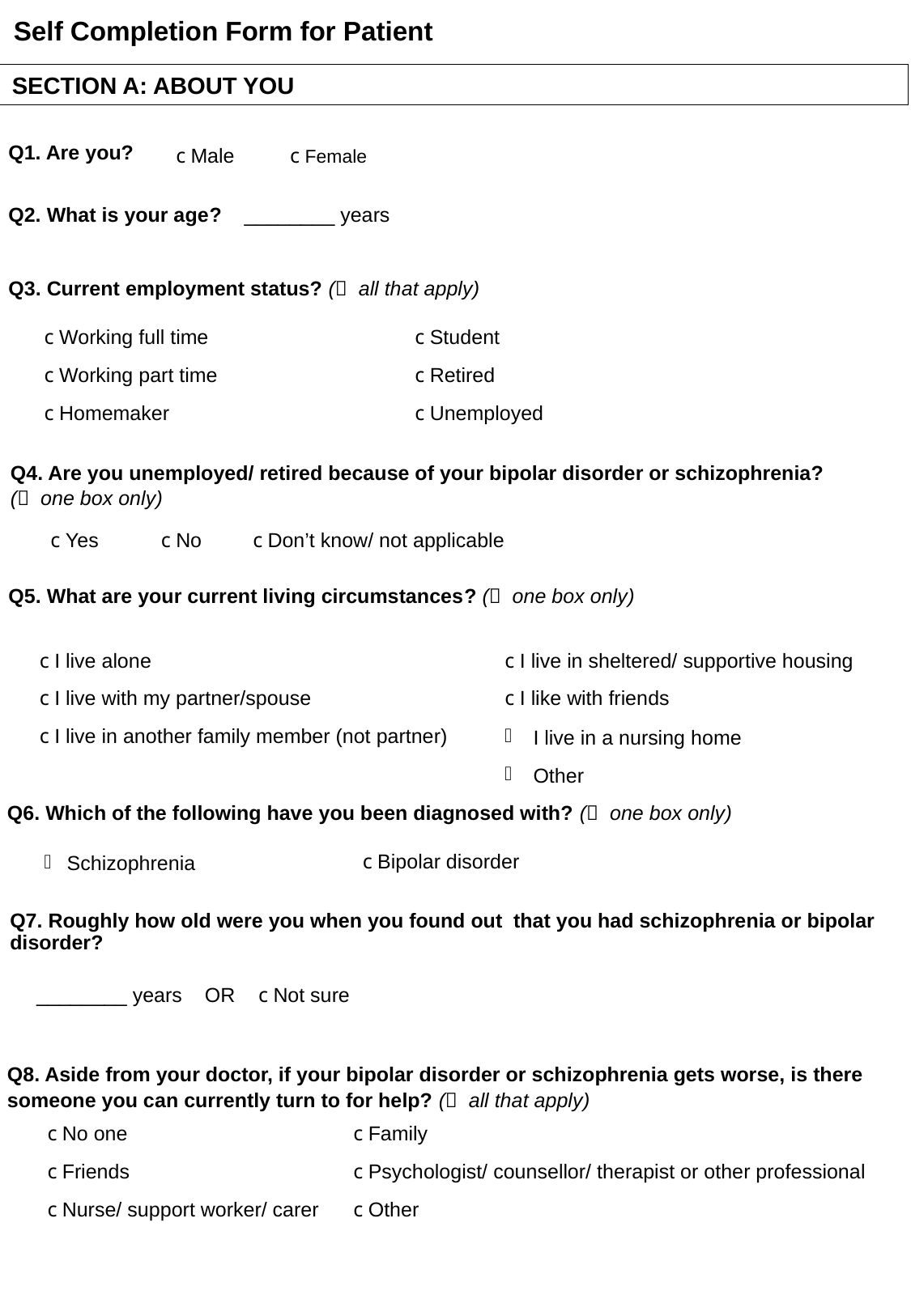

Self Completion Form for Patient
SECTION A: ABOUT YOU
| Q1. Are you? | c Male | c Female |
| --- | --- | --- |
| Q2. What is your age? \_\_\_\_\_\_\_\_ years |
| --- |
| Q3. Current employment status? ( all that apply) | | |
| --- | --- | --- |
| | c Working full time | c Student |
| | c Working part time | c Retired |
| | c Homemaker | c Unemployed |
| Q4. Are you unemployed/ retired because of your bipolar disorder or schizophrenia? ( one box only) | | |
| --- | --- | --- |
| | c Yes | c No c Don’t know/ not applicable |
| Q5. What are your current living circumstances? ( one box only) | | |
| --- | --- | --- |
| | c I live alone | c I live in sheltered/ supportive housing |
| | c I live with my partner/spouse | c I like with friends |
| | c I live in another family member (not partner) | I live in a nursing home |
| | | Other |
| Q6. Which of the following have you been diagnosed with? ( one box only) | | |
| --- | --- | --- |
| | Schizophrenia | c Bipolar disorder |
| Q7. Roughly how old were you when you found out that you had schizophrenia or bipolar disorder? \_\_\_\_\_\_\_\_ years OR c Not sure |
| --- |
| |
| Q8. Aside from your doctor, if your bipolar disorder or schizophrenia gets worse, is there someone you can currently turn to for help? ( all that apply) | | |
| --- | --- | --- |
| | c No one | c Family |
| | c Friends | c Psychologist/ counsellor/ therapist or other professional |
| | c Nurse/ support worker/ carer | c Other |

## Slide 3
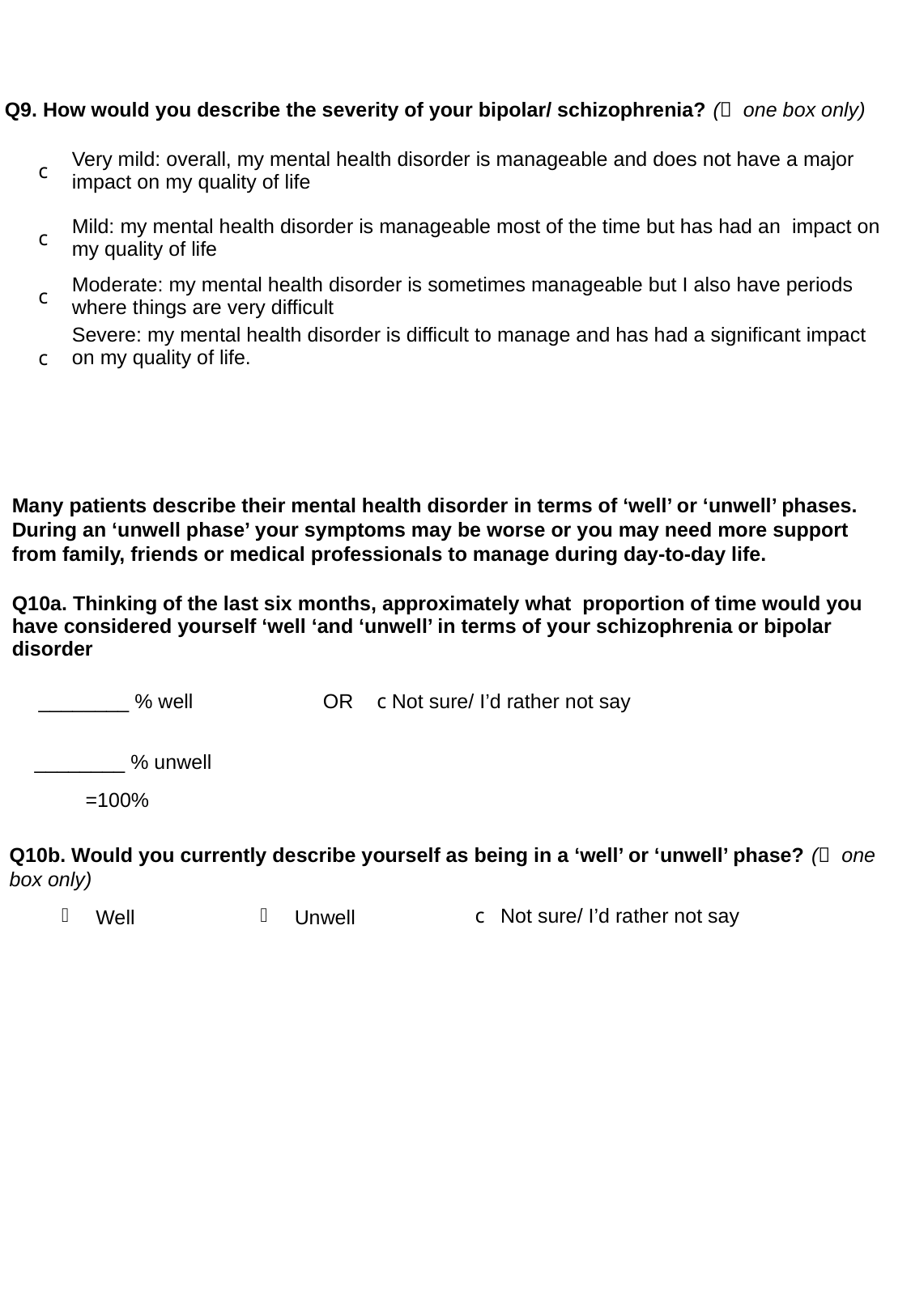

| Q9. How would you describe the severity of your bipolar/ schizophrenia? ( one box only) | | | |
| --- | --- | --- | --- |
| | c | Very mild: overall, my mental health disorder is manageable and does not have a major impact on my quality of life | |
| | c | Mild: my mental health disorder is manageable most of the time but has had an impact on my quality of life | |
| | c | Moderate: my mental health disorder is sometimes manageable but I also have periods where things are very difficult | |
| | c | Severe: my mental health disorder is difficult to manage and has had a significant impact on my quality of life. | |
Many patients describe their mental health disorder in terms of ‘well’ or ‘unwell’ phases. During an ‘unwell phase’ your symptoms may be worse or you may need more support from family, friends or medical professionals to manage during day-to-day life.
| Q10a. Thinking of the last six months, approximately what proportion of time would you have considered yourself ‘well ‘and ‘unwell’ in terms of your schizophrenia or bipolar disorder \_\_\_\_\_\_\_\_ % well OR c Not sure/ I’d rather not say |
| --- |
| \_\_\_\_\_\_\_\_ % unwell |
| =100% |
| Q10b. Would you currently describe yourself as being in a ‘well’ or ‘unwell’ phase? ( one box only) | | | |
| --- | --- | --- | --- |
| | Well | Unwell | c Not sure/ I’d rather not say |

## Slide 4
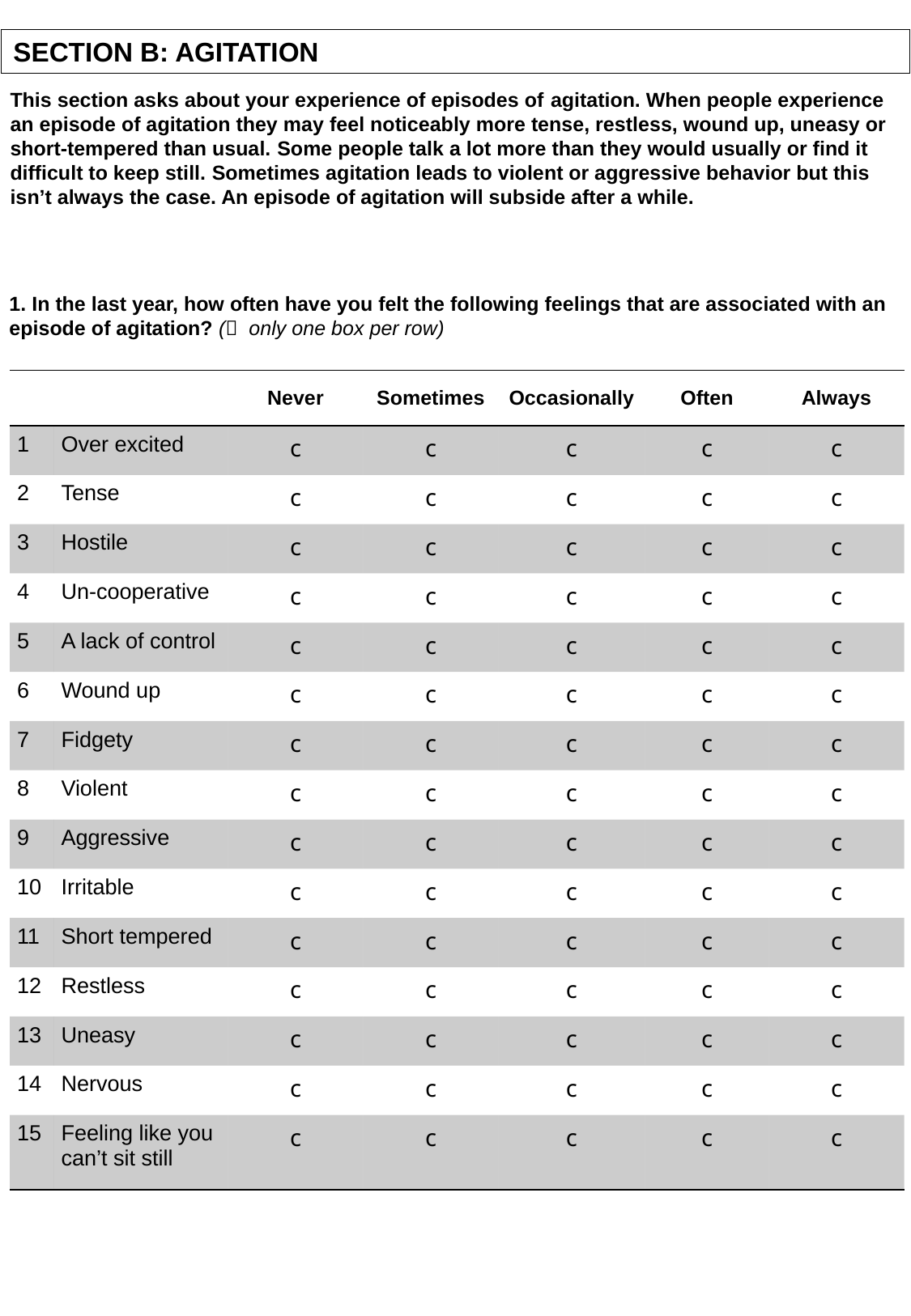

SECTION B: AGITATION
This section asks about your experience of episodes of agitation. When people experience an episode of agitation they may feel noticeably more tense, restless, wound up, uneasy or short-tempered than usual. Some people talk a lot more than they would usually or find it difficult to keep still. Sometimes agitation leads to violent or aggressive behavior but this isn’t always the case. An episode of agitation will subside after a while.
1. In the last year, how often have you felt the following feelings that are associated with an episode of agitation? ( only one box per row)
| | | Never | Sometimes | Occasionally | Often | Always |
| --- | --- | --- | --- | --- | --- | --- |
| 1 | Over excited | c | c | c | c | c |
| 2 | Tense | c | c | c | c | c |
| 3 | Hostile | c | c | c | c | c |
| 4 | Un-cooperative | c | c | c | c | c |
| 5 | A lack of control | c | c | c | c | c |
| 6 | Wound up | c | c | c | c | c |
| 7 | Fidgety | c | c | c | c | c |
| 8 | Violent | c | c | c | c | c |
| 9 | Aggressive | c | c | c | c | c |
| 10 | Irritable | c | c | c | c | c |
| 11 | Short tempered | c | c | c | c | c |
| 12 | Restless | c | c | c | c | c |
| 13 | Uneasy | c | c | c | c | c |
| 14 | Nervous | c | c | c | c | c |
| 15 | Feeling like you can’t sit still | c | c | c | c | c |

## Slide 5
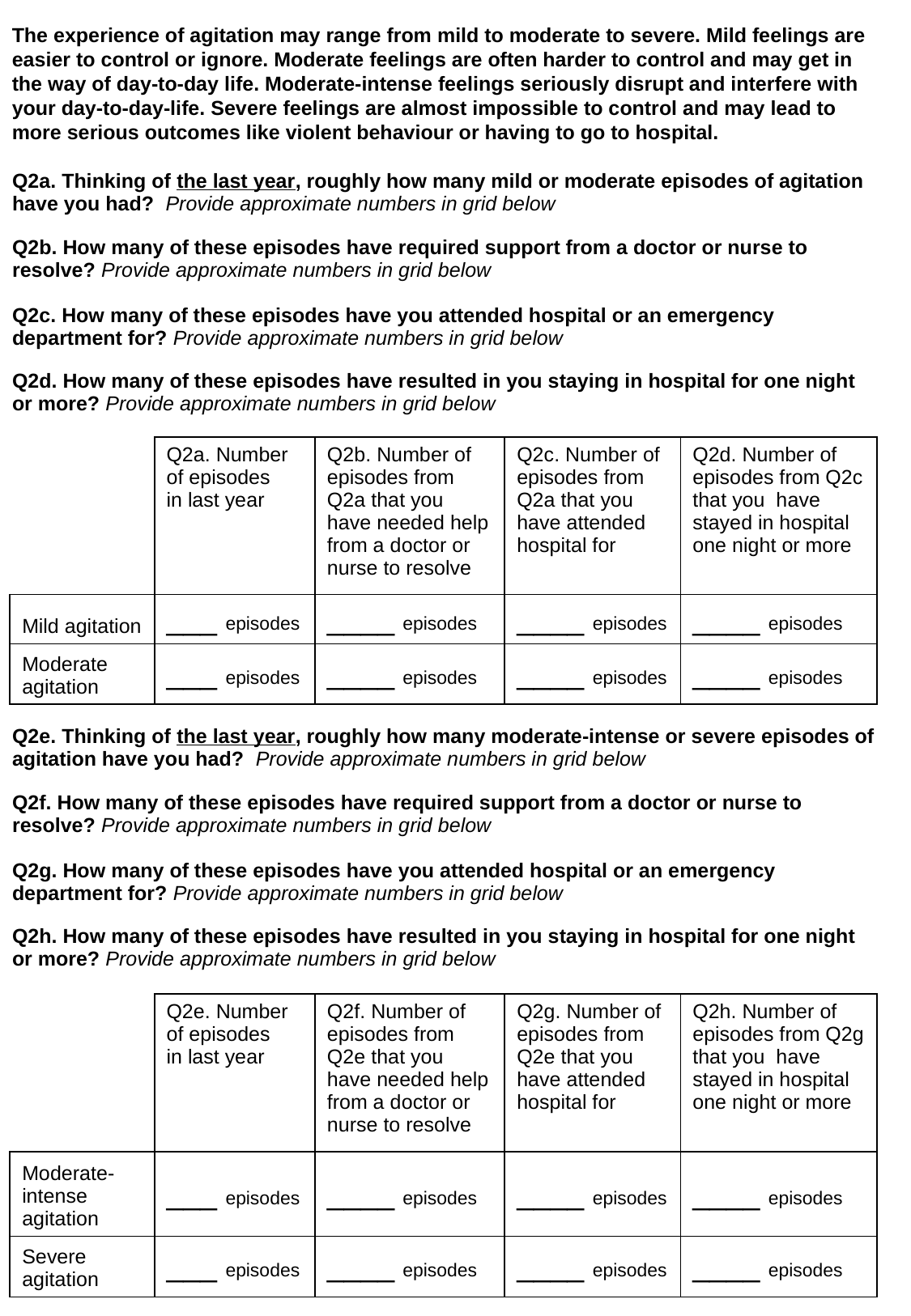

The experience of agitation may range from mild to moderate to severe. Mild feelings are easier to control or ignore. Moderate feelings are often harder to control and may get in the way of day-to-day life. Moderate-intense feelings seriously disrupt and interfere with your day-to-day-life. Severe feelings are almost impossible to control and may lead to more serious outcomes like violent behaviour or having to go to hospital.
| Q2a. Thinking of the last year, roughly how many mild or moderate episodes of agitation have you had? Provide approximate numbers in grid below |
| --- |
| Q2b. How many of these episodes have required support from a doctor or nurse to resolve? Provide approximate numbers in grid below Q2c. How many of these episodes have you attended hospital or an emergency department for? Provide approximate numbers in grid below |
| Q2d. How many of these episodes have resulted in you staying in hospital for one night or more? Provide approximate numbers in grid below |
| | Q2a. Number of episodes in last year | Q2b. Number of episodes from Q2a that you have needed help from a doctor or nurse to resolve | Q2c. Number of episodes from Q2a that you have attended hospital for | Q2d. Number of episodes from Q2c that you have stayed in hospital one night or more |
| --- | --- | --- | --- | --- |
| Mild agitation | \_\_\_ episodes | \_\_\_\_ episodes | \_\_\_\_ episodes | \_\_\_\_ episodes |
| Moderate agitation | \_\_\_ episodes | \_\_\_\_ episodes | \_\_\_\_ episodes | \_\_\_\_ episodes |
| Q2e. Thinking of the last year, roughly how many moderate-intense or severe episodes of agitation have you had? Provide approximate numbers in grid below |
| --- |
| Q2f. How many of these episodes have required support from a doctor or nurse to resolve? Provide approximate numbers in grid below Q2g. How many of these episodes have you attended hospital or an emergency department for? Provide approximate numbers in grid below |
| Q2h. How many of these episodes have resulted in you staying in hospital for one night or more? Provide approximate numbers in grid below |
| | Q2e. Number of episodes in last year | Q2f. Number of episodes from Q2e that you have needed help from a doctor or nurse to resolve | Q2g. Number of episodes from Q2e that you have attended hospital for | Q2h. Number of episodes from Q2g that you have stayed in hospital one night or more |
| --- | --- | --- | --- | --- |
| Moderate-intense agitation | \_\_\_ episodes | \_\_\_\_ episodes | \_\_\_\_ episodes | \_\_\_\_ episodes |
| Severe agitation | \_\_\_ episodes | \_\_\_\_ episodes | \_\_\_\_ episodes | \_\_\_\_ episodes |

## Slide 6
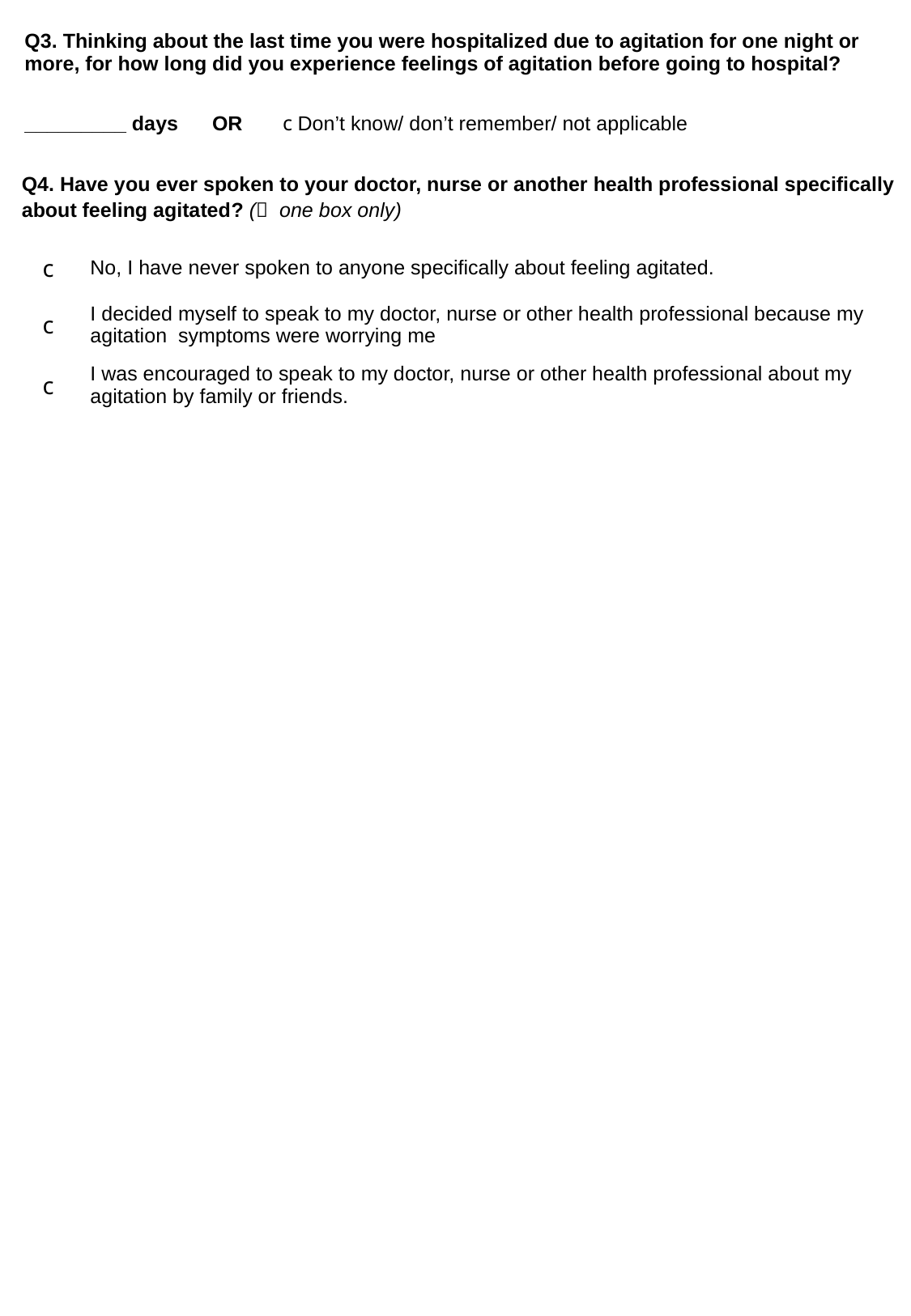

| Q3. Thinking about the last time you were hospitalized due to agitation for one night or more, for how long did you experience feelings of agitation before going to hospital? | |
| --- | --- |
| \_\_\_\_\_\_\_\_\_ days OR c Don’t know/ don’t remember/ not applicable | |
| Q4. Have you ever spoken to your doctor, nurse or another health professional specifically about feeling agitated? ( one box only) | | |
| --- | --- | --- |
| | c | No, I have never spoken to anyone specifically about feeling agitated. |
| | c | I decided myself to speak to my doctor, nurse or other health professional because my agitation symptoms were worrying me |
| | c | I was encouraged to speak to my doctor, nurse or other health professional about my agitation by family or friends. |

## Slide 7
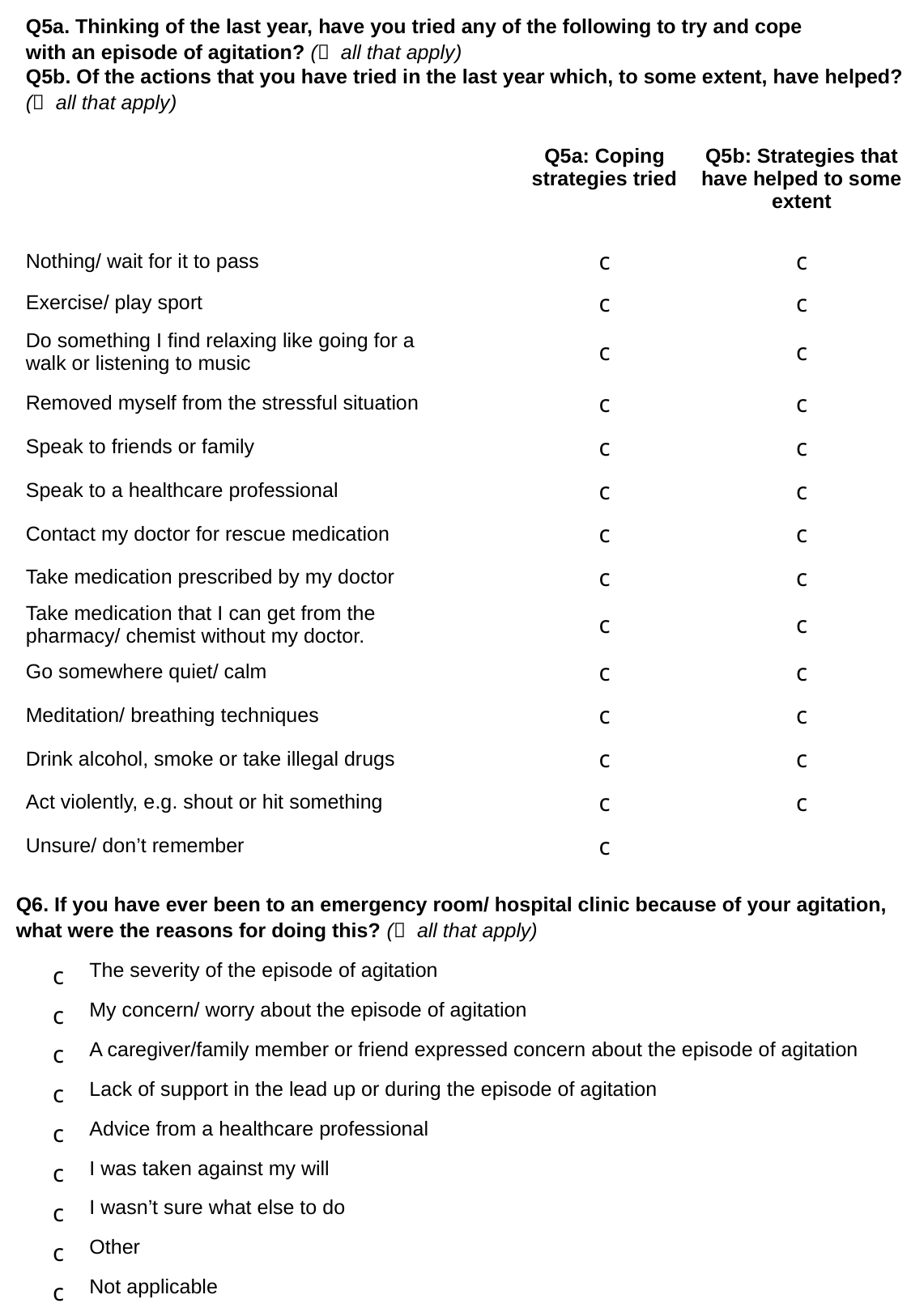

| Q5a. Thinking of the last year, have you tried any of the following to try and cope with an episode of agitation? ( all that apply) Q5b. Of the actions that you have tried in the last year which, to some extent, have helped? ( all that apply) | | |
| --- | --- | --- |
| | Q5a: Coping strategies tried | Q5b: Strategies that have helped to some extent |
| Nothing/ wait for it to pass | c | c |
| Exercise/ play sport | c | c |
| Do something I find relaxing like going for a walk or listening to music | c | c |
| Removed myself from the stressful situation | c | c |
| Speak to friends or family | c | c |
| Speak to a healthcare professional | c | c |
| Contact my doctor for rescue medication | c | c |
| Take medication prescribed by my doctor | c | c |
| Take medication that I can get from the pharmacy/ chemist without my doctor. | c | c |
| Go somewhere quiet/ calm | c | c |
| Meditation/ breathing techniques | c | c |
| Drink alcohol, smoke or take illegal drugs | c | c |
| Act violently, e.g. shout or hit something | c | c |
| Unsure/ don’t remember | c | |
| Q6. If you have ever been to an emergency room/ hospital clinic because of your agitation, what were the reasons for doing this? ( all that apply) | | |
| --- | --- | --- |
| | c | The severity of the episode of agitation |
| | c | My concern/ worry about the episode of agitation |
| | c | A caregiver/family member or friend expressed concern about the episode of agitation |
| | c | Lack of support in the lead up or during the episode of agitation |
| | c | Advice from a healthcare professional |
| | c | I was taken against my will |
| | c | I wasn’t sure what else to do |
| | c | Other |
| | c | Not applicable |

## Slide 8
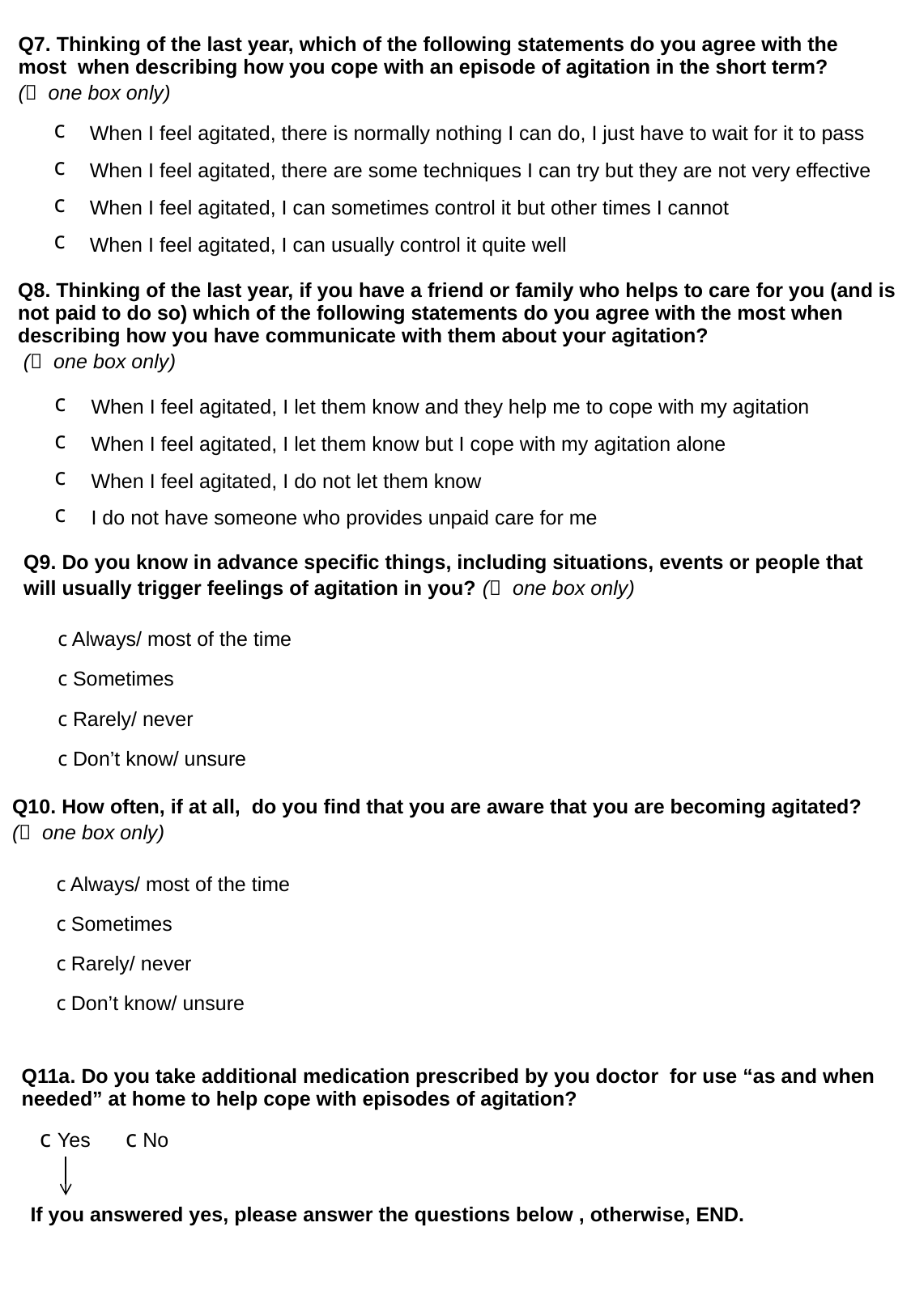

| Q7. Thinking of the last year, which of the following statements do you agree with the most when describing how you cope with an episode of agitation in the short term? ( one box only) | | |
| --- | --- | --- |
| | c | When I feel agitated, there is normally nothing I can do, I just have to wait for it to pass |
| | c | When I feel agitated, there are some techniques I can try but they are not very effective |
| | c | When I feel agitated, I can sometimes control it but other times I cannot |
| | c | When I feel agitated, I can usually control it quite well |
| Q8. Thinking of the last year, if you have a friend or family who helps to care for you (and is not paid to do so) which of the following statements do you agree with the most when describing how you have communicate with them about your agitation? ( one box only) | | |
| --- | --- | --- |
| | c | When I feel agitated, I let them know and they help me to cope with my agitation |
| | c | When I feel agitated, I let them know but I cope with my agitation alone |
| | c | When I feel agitated, I do not let them know |
| | c | I do not have someone who provides unpaid care for me |
| Q9. Do you know in advance specific things, including situations, events or people that will usually trigger feelings of agitation in you? ( one box only) | |
| --- | --- |
| | c Always/ most of the time |
| | c Sometimes |
| | c Rarely/ never |
| | c Don’t know/ unsure |
| Q10. How often, if at all, do you find that you are aware that you are becoming agitated? ( one box only) | |
| --- | --- |
| | c Always/ most of the time |
| | c Sometimes |
| | c Rarely/ never |
| | c Don’t know/ unsure |
| Q11a. Do you take additional medication prescribed by you doctor for use “as and when needed” at home to help cope with episodes of agitation? |
| --- |
| c Yes c No |
If you answered yes, please answer the questions below , otherwise, END.

## Slide 9
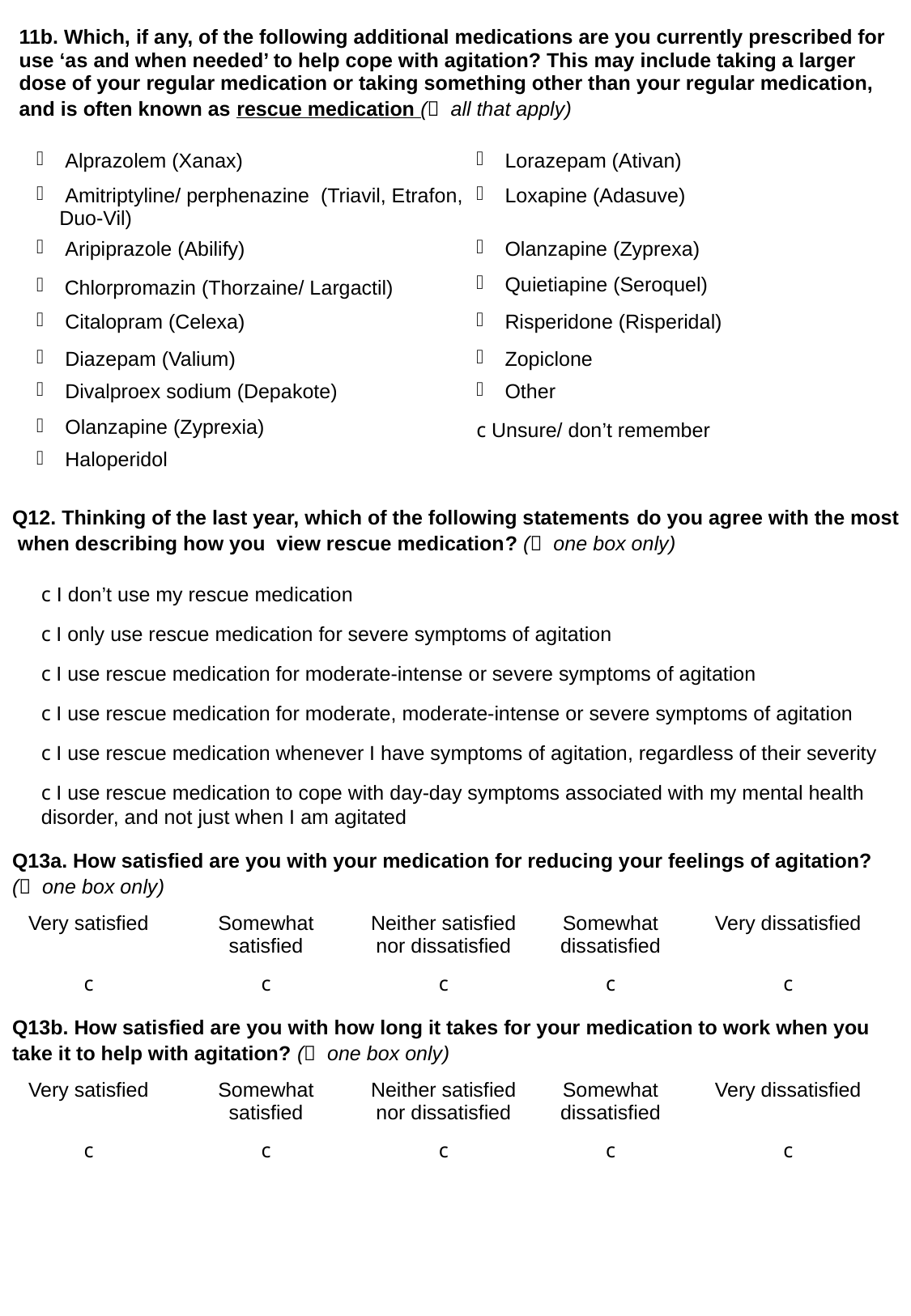

| 11b. Which, if any, of the following additional medications are you currently prescribed for use ‘as and when needed’ to help cope with agitation? This may include taking a larger dose of your regular medication or taking something other than your regular medication, and is often known as rescue medication ( all that apply) | | |
| --- | --- | --- |
| | Alprazolem (Xanax) | Lorazepam (Ativan) |
| | Amitriptyline/ perphenazine (Triavil, Etrafon, Duo-Vil) | Loxapine (Adasuve) |
| | Aripiprazole (Abilify) | Olanzapine (Zyprexa) |
| | Chlorpromazin (Thorzaine/ Largactil) | Quietiapine (Seroquel) |
| | Citalopram (Celexa) | Risperidone (Risperidal) |
| | Diazepam (Valium) | Zopiclone |
| | Divalproex sodium (Depakote) | Other |
| | Olanzapine (Zyprexia) | c Unsure/ don’t remember |
| | Haloperidol | |
| Q12. Thinking of the last year, which of the following statements do you agree with the most when describing how you view rescue medication? ( one box only) | |
| --- | --- |
| | c I don’t use my rescue medication |
| | c I only use rescue medication for severe symptoms of agitation |
| | c I use rescue medication for moderate-intense or severe symptoms of agitation |
| | c I use rescue medication for moderate, moderate-intense or severe symptoms of agitation |
| | c I use rescue medication whenever I have symptoms of agitation, regardless of their severity |
| | c I use rescue medication to cope with day-day symptoms associated with my mental health disorder, and not just when I am agitated |
| Q13a. How satisfied are you with your medication for reducing your feelings of agitation? ( one box only) | | | | |
| --- | --- | --- | --- | --- |
| Very satisfied | Somewhat satisfied | Neither satisfied nor dissatisfied | Somewhat dissatisfied | Very dissatisfied |
| c | c | c | c | c |
| Q13b. How satisfied are you with how long it takes for your medication to work when you take it to help with agitation? ( one box only) | | | | |
| --- | --- | --- | --- | --- |
| Very satisfied | Somewhat satisfied | Neither satisfied nor dissatisfied | Somewhat dissatisfied | Very dissatisfied |
| c | c | c | c | c |

## Slide 10
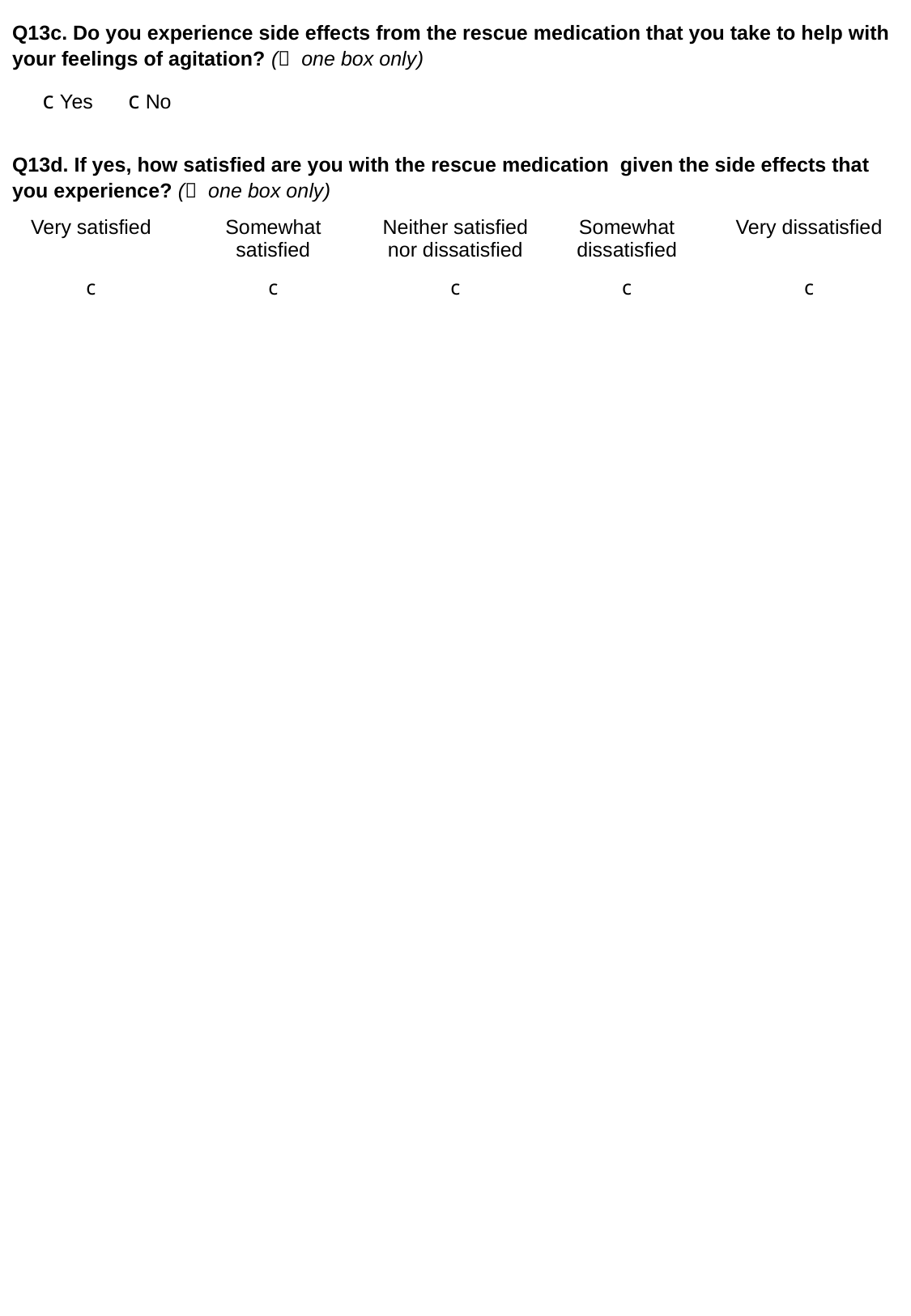

| Q13c. Do you experience side effects from the rescue medication that you take to help with your feelings of agitation? ( one box only) | | | | |
| --- | --- | --- | --- | --- |
| c Yes c No | | | | |
| Q13d. If yes, how satisfied are you with the rescue medication given the side effects that you experience? ( one box only) | | | | |
| Very satisfied | Somewhat satisfied | Neither satisfied nor dissatisfied | Somewhat dissatisfied | Very dissatisfied |
| c | c | c | c | c |
